# Investigating the Role of Mitochondrial DNA in Alzheimer’s Disease Biomarker Expression

**DOI:** 10.1002/alz.092424

**Published:** 2025-01-03

**Authors:** Riley E Kemna, Ian Weidling, Paul J Kueck, Chelsea N Johnson, Casey S. John, Russell H Swerdlow, Heather M Wilkins, Jill K Morris

**Affiliations:** ^1^ University of Kansas Medical Center, Kansas City, KS USA; ^2^ University of Kansas Alzheimer’s Disease Research Center, Fairway, KS USA; ^3^ AbbVie, Cambridge, MA USA; ^4^ University of Kansas Alzheimer’s Disease Center, Fairway, KS USA

## Abstract

**Background:**

Impaired metabolic function and mitochondrial metabolism increase risk of Alzheimer’s Disease (AD) development, which is the leading form of dementia and one of the main causes of death in older adults. Altered mitochondrial function can reduce efficiency of cellular maintenance processes like mitophagy and proteostasis, leading to protein aggregation and cytotoxicity. Mitochondria differ from other organelles, as they have their own unique genetic component (mtDNA), which encodes proteins essential for mitochondrial translation and oxidative metabolism. Differences in mtDNA between individuals affect mitochondrial function and can increase risk of certain diseases; it is not well studied how mtDNA impacts AD pathology.

**Method:**

SH‐SY5Y cytoplasmic hybrid (cybrid) cell lines were generated using mtDNA from clinical research volunteers (n = 18 cognitively healthy (CH) older adults (mean age 73.8), n = 7 MCI (mean age 78.1), n = 10 AD (mean age 75.3)) enrolled in the Relationship of Energetics and Cognitive Trajectory study. Groups did not differ by sex. Cells were analyzed for protein expression by western blot, metabolic flux by Agilent Seahorse XF Analyzer, and protein secretion by ELISA. Plasma pTau217 (AlzPATH) and Aβ42 (N4PE) were assessed by Simoa HD‐X (Quanterix) to compare blood biomarker values with cellular outcomes.

**Result:**

Cybrids from individuals with MCI and AD had elevated intracellular pTau217 (p = 0.033, p = 0.006 respectively). Cybrids from CH individuals secreted significantly more Aβ42 than those from individuals with AD (p = 0.002). Cybrid pTau217 and plasma pTau217 from the same subjects correlated significantly (p<0.001). Cybrids showed altered mitochondrial function by diagnostic group in all ETC complexes (p<0.05), and altered ETC complex expression in all proteins except Complex II, which is not encoded by mtDNA. Mitochondrial transcription factor A (TFAM) was significantly reduced in cybrids from AD individuals (p = 0.010) compared to those from CH subjects.

**Conclusion:**

Cytoplasmic hybrid cell lines generated with mtDNA from individuals with and without AD express diagnostic differences in novel AD biomarkers. This suggests that mitochondrial function, specifically that which is influenced by mtDNA, plays an imperative role in AD pathology. Ongoing research in the cybrid cell model will help elucidate how altered cellular and bioenergetic function contribute to AD etiology.

